# Predation has a significant impact on the complexity and stability of microbial food webs in subalpine lakes

**DOI:** 10.1128/spectrum.02411-23

**Published:** 2023-10-03

**Authors:** Ping Guo, Cui Li, Jinxain Liu, Baofeng Chai

**Affiliations:** 1 Shanxi Key Laboratory of Ecological Restoration on Loess Plateau, Institute of Loess Plateau, Shanxi University, Taiyuan, China; 2 Central Laboratory, Changzhi Medical College, Changzhi, China; 3 Faculty of Environment Economics, Shanxi University of Finance and Economics, Taiyuan, China; Oklahoma State University, Stillwater, Oklahoma, USA

**Keywords:** microbial food webs, complexity and stability, protist communities, predation

## Abstract

Microbial food webs are drivers of material circulation and energy flow in lake ecosystems. The structural shifting of microbial food webs has important implications for the function of lake ecosystems. However, we continue to lack a clear understanding of the mechanisms underlying the complexity and stability of microbial food web structures. In this study, we investigated the diversity and structure of protist communities and bacterial communities by using DNA meta-barcoding to reveal the key factors affecting the complexity and stability of microbial food webs in subalpine lakes. Our analysis shows that significant seasonal variations occurred in protist taxonomic and functional communities (e.g., algivores, bacterivores, and phototrophs), which were mainly controlled by hydrological and nutrient parameters such as temperature, pH, and NH_4_^+^-N. Based on the network approach, the most complex food webs in the summer exhibited the lowest stability. We also found that the trophic interaction between bacterivorous protozoans and bacteria was the main factor significantly having influence on microbial food web complexity and stability (*P* < 0.01). Moreover, biotic factors (i.e., bacterivorous protozoan composition) were better predictors of the relative abundance of several bacteria (Proteobacteria and Cyanobacteria) as prey than abiotic factors. Thus, these results suggest that predation is essential for the complexity and stability of the microbial food webs in lake ecosystems. Our findings suggest that understanding these complex interactions of microbial food webs is critical for ecosystem management and for predicting the ecological consequences of future climate changes in aquatic ecosystems.

**IMPORTANCE:**

As an important part of microbial food webs, protists transfer organic carbon and nutrients to higher trophic levels in aquatic ecosystems. Protist predation often influences the abundance and composition of bacterial communities. However, we still do not understand whether and how predation affects the complexity and stability of microbial food webs. This study assessed the seasonal dynamic characteristics and driving factors of microbial food webs in terms of complexity and stability. Our findings have implications for future surveys to reveal the effects of climate and environmental changes.

## INTRODUCTION

Microbial food webs play an important role in material recycling and energy flow in natural ecosystems (1, 2) and maintain the structural and functional stability of these ecosystems through interactions among different trophic levels (3). Microbial food webs are always in dynamic equilibrium, and self-regulation induced by environmental factors (e.g., temperature and nitrate) can lead to changes in the webs’ structure and function (4 – 6). Climate change enhances the complexity and stability of microbial networks (7), which exhibit distinct seasonal dynamics (8). On the other hand, model experiments show that the mixotrophic interactions of microbial food webs may enhance network stability (9). The functional responses of microbial food webs to ecological stress are reflected in changes to the complexity and stability of ecosystem function. Therefore, understanding the maintenance mechanism of the stability and complexity of microbial food webs is critical for elucidating ecosystem functions in the context of global climate change.

Many factors influence the complexity and stability of food webs. Notably, the habitat type plays a vital role in shaping the structure and stability of Antarctic food webs (10), whereas temperature was previously believed to have a positive correlation with food web complexity (connectivity) and omnivory and trophic levels (11, 12). Climate change and anthropogenic activities have led to more complex food webs in the Beagle Channel; however, the food webs are less stable than those in Potter Cove (13). As biotic factors, generalist top predators have enhanced the stability of food webs (14), while trophic coherence has been a determining factor for food web stability (15). Despite extensive research on the relationships between complexity and stability, the definition and research methods remain controversial. With the continuous development of research methods and technologies, great progress has been made in the research of microbial food webs at the microscale. Microbial food webs describe the trophic interactions among alga, heterotrophic bacteria, and protozoa—including flagellates and ciliates (5). In aquatic ecosystems, the material and energy of microbial food webs flow from primary to higher trophic levels and finally enter the classic food web (5). Therefore, microbial food webs are a material and energy source in lake water ecosystems, and their structure and function are crucial for maintaining water ecological health (16).

As an important part of microbial food webs, protists participate in numerous essential ecological and biogeochemical cycles in aquatic and terrestrial environments (17). Protists have a variety of trophic modes, including autotrophic (autotrophs, often referred to as algae), heterotrophic (heterotrophs, often referred to as protozoa), and mixotrophic. As primary producers, autotrophic protists are recognized as major contributors to biomass, primary production, and respiration (18, 19). As predators, heterotrophic protists feeding on phytoplankton and heterotrophic microorganisms (mainly bacteria and fungi) are major consumers in microbial food webs and transfer organic carbon and nutrients to higher trophic levels. Simultaneously, heterotrophic protists, as a food source for metazoan zooplankton, play a vital role in marine ecosystems (18). The composition of protist communities is more sensitive to environmental changes and human activities in aquatic ecosystems (20, 21). Recent evidence indicates that biotic (e.g., predators) and abiotic (e.g., temperature) factors play a crucial role in shaping the structure of protist communities (22). However, knowledge of the key factors influencing the structure of protist communities, especially functional communities, remains limited, hindering our understanding of their ecological role and environmental relevance. Predation is dominated by phagotrophic protists in aquatic microbial food webs; however, the roles of these microorganisms in ecosystems remain understudied when compared with those of phytoplankton and bacteria (23). Moreover, previous studies have mostly focused on the composition of protist communities and the distribution patterns of species diversity. In recent years, the predator‒prey relationships and functions of protozoa in microbial food webs have gradually become recognized. Ciliates prey on heterotrophic flagellates to control energy transfer within microbial food webs (24). The feeding of heterotrophic dinoflagellates affects bacterial species richness and alters energy flow processes in marine environments (25). The predation of protists on bacterial communities profoundly affects their structure and function (26). Moreover, predator richness increases prey diversity in microcosm experiments (25). Predation appears to play a key role in structuring microbial food webs. Protistan grazing can selectively reduce the relative abundance of some bacterial groups, such as unicellular cyanobacteria or specific freshwater β-proteobacteria (27). Predatory protists strongly influence bacterial abundance, diversity, and productivity (27 – 29), but it remains uncertain whether and how they affect microbial food web complexity and stability.

Previous studies of microbial food webs have mainly been conducted on oceans (2, 30), lowland lakes (16), and rivers (22). Most alpine lakes are relatively closed ecosystems and particularly sensitive to climate change and human interference (31). The food webs of alpine lakes are relatively simple and can respond more rapidly and sensitively to changes in the environment, which is especially true for extreme environmental conditions (e.g., strong radiation, low temperature, and the input of many nutrients) (32). In our previous investigations, a high diversity of the bacterial community during ice melting (33) and a clear difference in phytoplankton community structure (31) in subalpine lakes were observed. However, we still lack an understanding of the maintenance mechanism of the stability and complexity of microbial food webs in lake ecosystems. In this study, we conducted an investigation of the microbial food webs of bacteria and protists in a natural subalpine lake by using 16S and 18S rRNA gene sequencing to address the following questions: (i) What are the main factors affecting the diversity and structure of protist communities and functional communities in a subalpine lake? (ii) What are the seasonal dynamic characteristics of microbial food webs in terms of complexity and stability? (iii) What are the most important factors affecting the structural complexity and stability of microbial food webs?

## RESULTS

### Seasonal dynamics of protist communities

**Fig 1:**
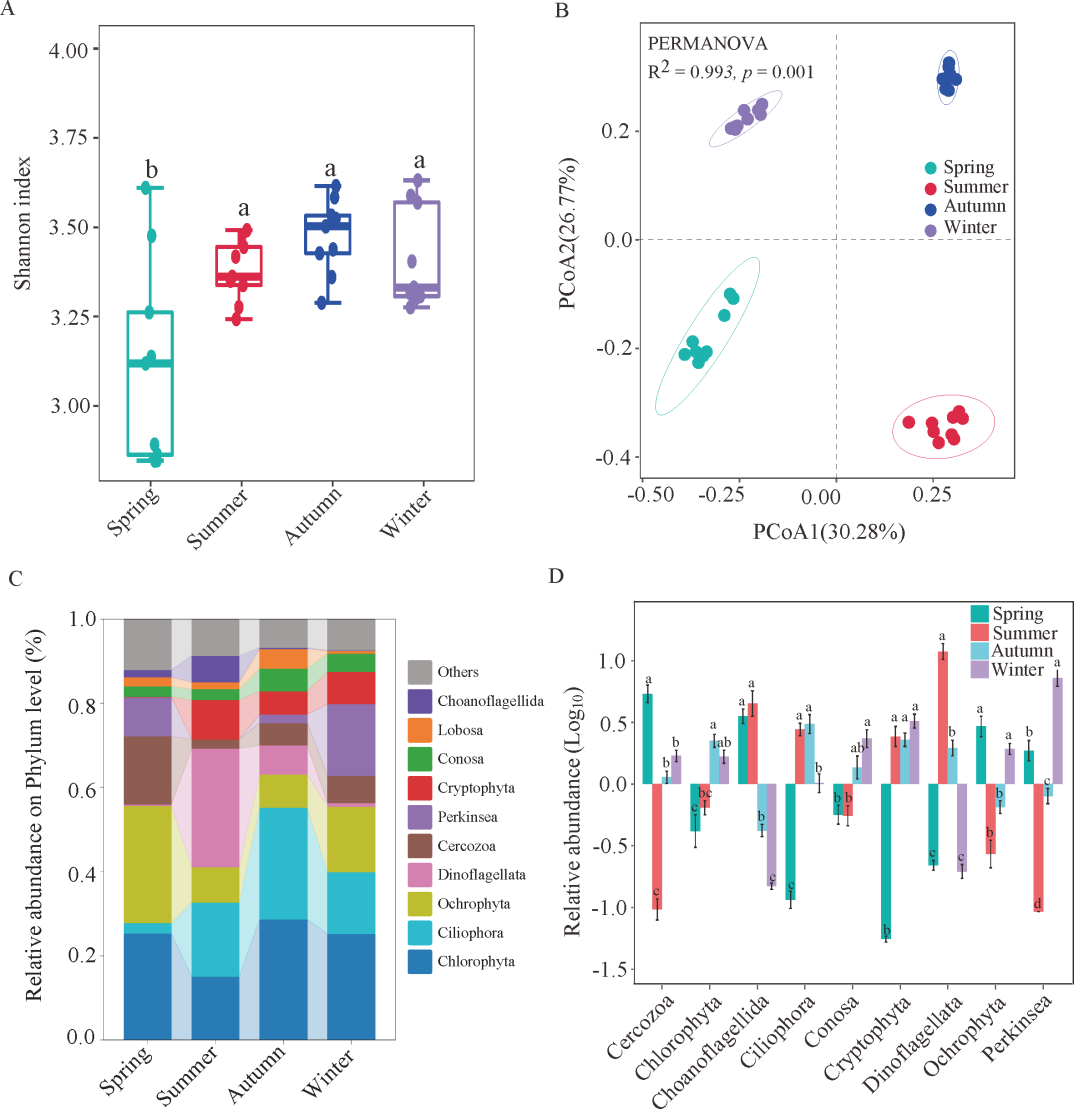
The diversity and community composition of protists in different seasons. (**A**) Alpha diversity estimates (Shannon index). (**B**) PCoA analysis based on Bray‒Curtis distance. (**C**) Relative abundance of dominant protist phyla. (**D**) Phylum-level species for significance test analysis of differences among the four seasons. A difference in letters indicates a significant difference (*P* < 0.05).

To study the seasonal dynamics of diversity, sequences were clustered into different OTUs according to a sequence similarity of 97%, and the alpha diversity of all protists was calculated based on each sample’s OTUs. The diversity index significantly differed in different seasons (*P* < 0.001), while the Shannon index in spring was lower than that in the other three seasons (Fig. 1A). The Spearman correlation analysis indicated that the Shannon index of protist communities was mainly affected by temperature, SAL, and DO (Fig. S2A). The PCoA revealed remarkable seasonal variations in the composition of protist communities. PERMANOVA (*R* ^2^ = 0.993, *P* < 0.001) further revealed that protist communities were significantly different during the four seasons (Fig. 1B). Redundancy analysis (RDA) showed that the composition of protist communities was mainly influenced by SAL (explaining 15.11% of total variation), NH_4_^+^-N (13.65%), temperature (13.16%), SO_4_^2−^-S (12.89%), IC (12.79%), TOC (10.60%), DO (8.06%), EC (6.89%), and NO_3_^−^-N (6.81%) (*F* = 2.2644, *P* < 0.05). The first two axes explained 53.53% of the structural variability of protist communities (Fig. S2B).

Protists were the most dominant eukaryotes in the subalpine lake, representing 73%, 70.7%, 67.7%, and 67.3% of the total eukaryotic reads in different seasons (Fig. S3). Stramenopiles were the most abundant protist supergroup in spring, followed by Archaeplastida, Rhizaria, Alveolata, and Amoebozoa (Fig. S3). To further analyze changes in the composition of protist communities, the relative abundance in different seasons was determined at the phylum level. Our results indicated that the protist communities were dominated by Chlorophyta (23.54%), Ciliophora (15.36%), and Ochrophyta (14.91%) (Fig. 1C). One-way ANOVA revealed that there was significant seasonal variation in most phyla in terms of relative abundance (*P* < 0.05), except in Lobosa (*P* > 0.05) (Fig. 1D).

### Seasonal dynamics of functional protistan communities

**Fig 2:**
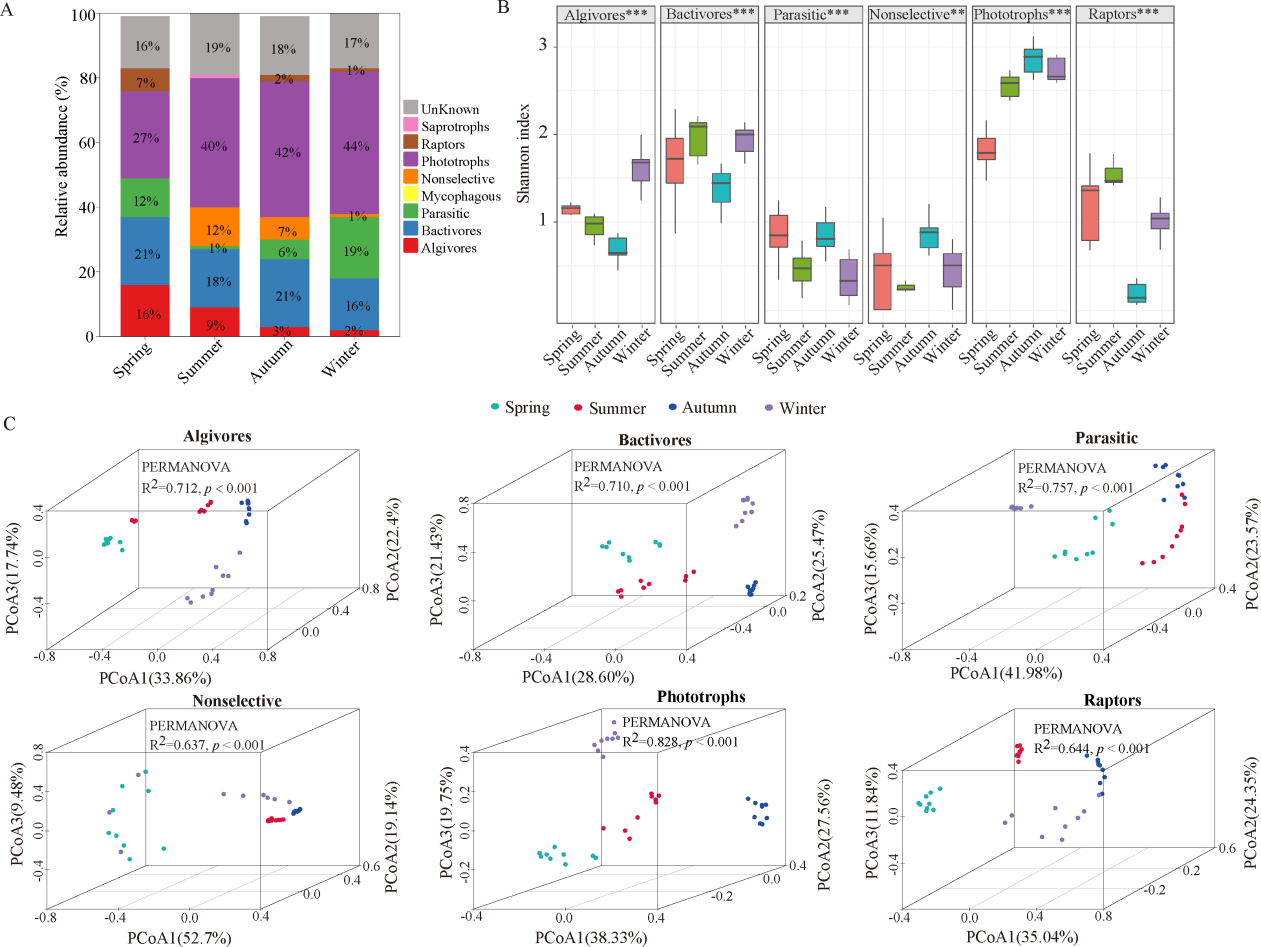
Diversity and composition of protistan functional communities in different seasons. (**A**) The composition of protistan functional communities. (**B**) Alpha diversity estimates (Shannon index). (**C**) PCoA based on Bray‒Curtis distance.

The protistan communities were functionally divided into algivores, bacterivores, parasites, mycophagous, nonselective omnivores, phototrophs, raptors, saprotrophs, and unknown (Fig. 2A; Table S2). The most abundant protistan functional communities in all seasons were phototrophs, followed by bacterivores, parasites, algivores, nonselective omnivores, and raptors (Fig. 2A). The alpha diversities (Shannon index) of all functional groups showed significant differences during the four seasons (*P* < 0.05), where the phototroph functional group had the highest biodiversity (Fig. 2B; Fig. S4A). The PCoA analysis showed that protistan functional groups (algivores, bacterivores, parasites, nonselective omnivores, phototrophs, and raptors) could be clearly divided into four communities according to seasonal changes (Fig. 2C). The results of the PERMANOVA revealed that there were seasonal differences in protistan functional community structures (*P* < 0.001) (Fig. 2C), suggesting that the functional groups of protists were affected by environmental factors. Mantel test analysis was used to evaluate the relationships of environmental factors and the structures of the protistan functional groups (Fig. S5). The results suggested that the functional groups of protists were significantly affected by many environmental factors (*P* < 0.05), except PO_4_^3−^ (*P* > 0.05).

Further analysis was conducted to illustrate the communities’ functional composition at the genus level (Fig. S6). The relative abundance of algivores gradually decreased with seasonal variation, with the genera *Askenasia*, *Chrysochromulina*, *Litostomatea_XXX*, *Ochromonas*, *Rhogostoma-lineage_X*, and *unclassified_o__Pterocystida* being significantly concentrated in different seasons (*P* < 0.05) (Fig. 2A; Fig. S6A). The relative abundance of 10 bacterivore genera (*Vorticella*, *Flamella*, *Spumella*, *Halteriidae_X*, *Rhogostoma-lineage_X*, *Chrysochromulina*, *Uroleptus*, *Sandona*, *Litostomatea_XXX*, and *unclassified_o__Pterocystida*) varied significantly between the four seasons (*P* < 0.05) (Fig. S6B). The parasitic group was more abundant in winter (Fig. 2A), while the genera *Peronosporales_X* and *Hartmannella* were enriched in the spring and summer, respectively (Fig. S6C). Nearly all of the phototrophic genera varied significantly in the four seasons, except for *Parvodinium* and *Katablepharidales_XX* (*P* < 0.05) (Fig. S6D). The genera *Litostomatea_XXX*, *Rhogostoma-lineage_X*, *Protaspa-lineage_X*, and *unclassified_o__Pterocystida*, which belong to the Centroheliozoa, Cercozoa, and Ciliophora in raptor groups, were enriched during the spring and summer (Fig. S6E).

### Microbial food webs construction and topological properties

**Fig 3:**
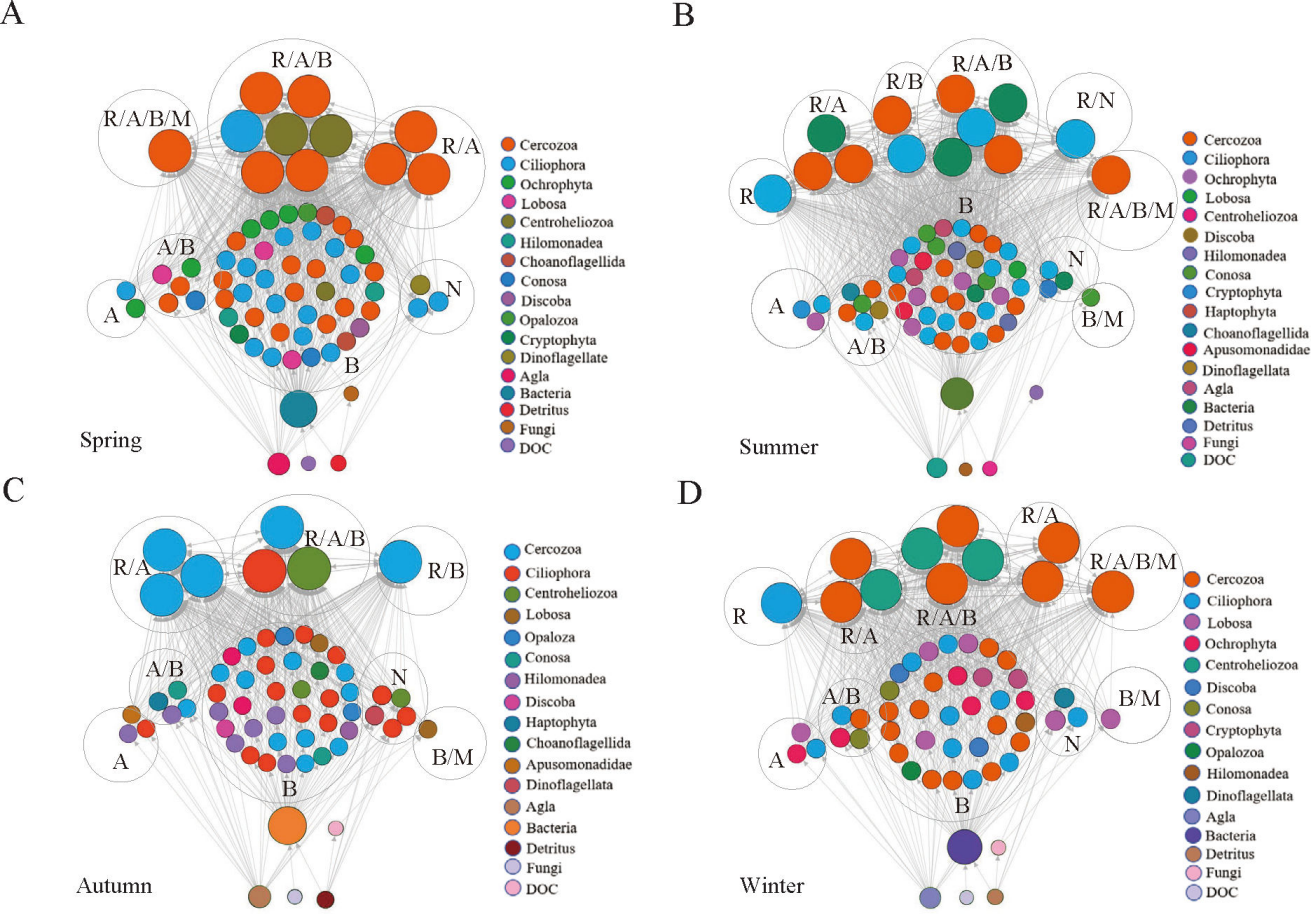
Graphic representation of microbial food webs in different seasons. (**A**) Spring, (**B**) summer, (**C**) autumn, and (**D**) winter. Each node represents a genus, algae, bacteria, detritus, fungi, or dissolved organic carbon (DOC). The size of the node represents the number of connections between nodes; node colors represent different phyla, while the arrow direction represents the predation relationship between different genera. The subtitles in circles indicate the different functional groups to which they belong. The letters in circles A, B, M, N, and R represent algivores, bacterivores, mycophagous, nonselective omnivores, and raptors, respectively.

Based on the relationship between predators and prey, the microbial food webs in the subalpine lake were constructed using a network approach (Fig. 3; Table S2). To evaluate the structural properties of seasonal microbial food webs, we calculated food web metrics (Table S8). The number of total species representing predators and prey in the four seasons was 69, 70, 61, and 59. The main properties of complexity included links (L) (780, 921, 462, and 646), linkage density (LD) (11.30, 13.20, 7.57, and 10.94), and connectance (C) (0.16, 0.19, 0.12, and 0.16) in the four seasons. The results revealed that the microbial food webs in summer were the most complex networks. Moreover, the clustering coefficients (CCs) of the summer (0.43) and winter (0.43) were higher than those of the spring (0.39) and autumn (0.29), while the characteristic path length (CPL) was the shortest in the summer. The findings also indicated that the percentage of bacterivore protistans (e.g., Cercozoa and Ciliophora) accounted for 45%–78% among all predators, indicating that they played a greater role in microbial food webs (Fig. 3; Fig. S4B). Overall, these topological properties indicated that the microbial food webs in different seasons differed with regard to structural and complexity properties (Fig. 3).

**Fig 4:**
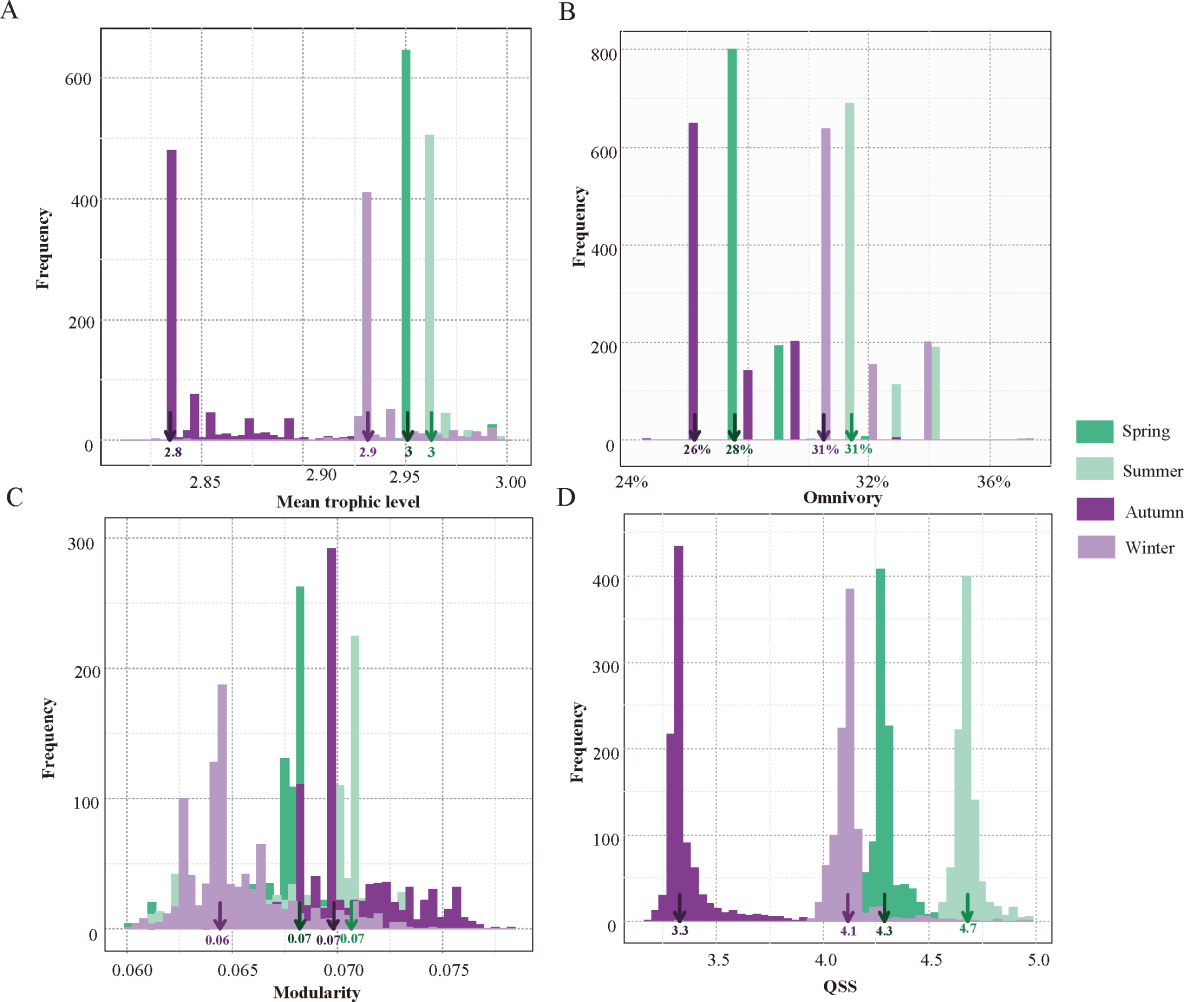
Randomization algorithm for the structure and stability of microbial food webs. (**A**) Mean trophic level, (**B**) omnivory, (**C**) modularity, and (**D**) QSS histograms of simulated networks were generated for the different microbial food webs. The arrows represent the empirical metric values, while the value is below each arrow.

To evaluate the structure and stability of microbial food webs, we calculated the mean trophic level (mean TL), omnivory (O), modularity (Mod), and quasi sign-stability (QSS). The results suggest that the metrics of structure and stability differed statistically (*P* < 0.01) among seasons (Table S9). Notably, all empirical values for microbial food webs were within the distributions of the simulated networks (Table S10). In spring, summer, autumn, and winter, the mean TLs were 2.8, 2.9, 3, and 3, respectively, and the Mod indices were 0.06, 0.07, 0.07, and 0.07 (Fig. 4A and C), respectively. The percentage of omnivory was higher in summer and winter (31% and 31%, respectively) than in spring and autumn (26% and 28%, respectively) (Fig. 4B). Moreover, QSS showed significant differences in microbial food webs between the four seasons (*P* < 0.01) (Fig. 4D). The QSS of microbial food webs in summer also presented a higher value (4.7); however, autumn had the lowest value (3.3), indicating that the probability of recovery in autumn was higher after perturbation.

### Drivers of complexity and stability in microbial food webs

This study focused on the primary components of the microbial food webs, including bacterivore protozoans, which displayed the highest percentage among all predators (45%–78%) (Fig. 3; Fig. S4B), and bacteria. Random forest (RF) analysis indicated that the diversity (richness) and composition (PCoA1) of bacterivorous protozoans were important predictors influencing the complexity metrics (L, LD, and C) and stability metrics (mean TL, O, Mod, and QSS) of microbial food webs (Fig. S7). We further tested the relationship between the complexity and stability of microbial food webs. All of the complexity metrics were significantly and positively correlated with the mean TL and QSS of stability metrics; however, they were significantly and negatively correlated with modularity and not correlated with omnivory (Table S10).

**Fig 5:**
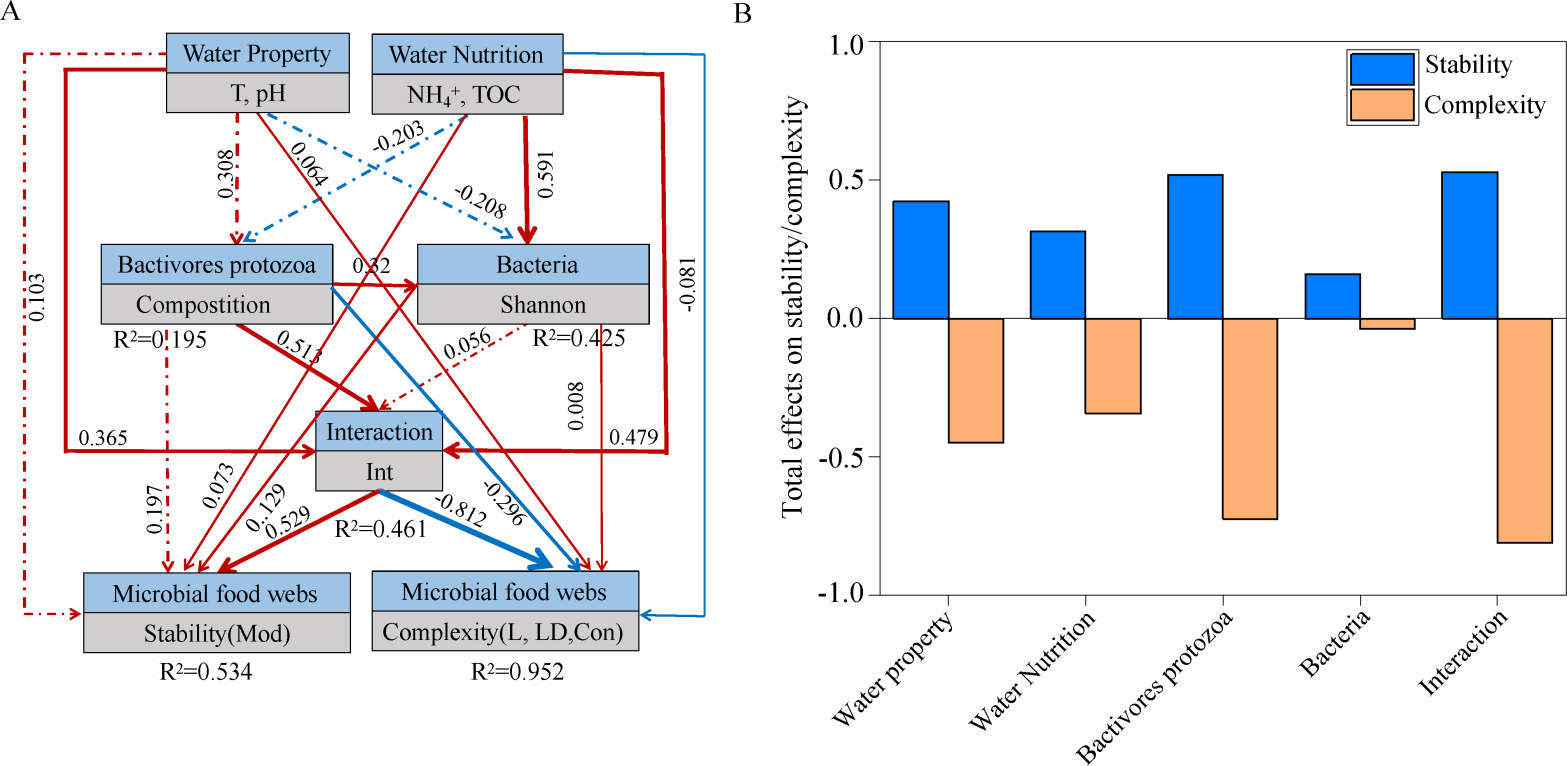
Contribution of abiotic and biotic factors to microbial food web complexity and stability. (**A**) PLS‐PM shows the direct and indirect effects. (**B**) Standard effects of the latent variables on microbial food web complexity and stability.

This study calculated the topological features of each sub-network by preserving the nodes of each sample. Biotic factors were inferred by calculating the average degree (AD) and the proportion of interaction associations between bacterivorous protozoans and bacterial taxa (Int). To explore the direct and indirect effects on the complexity and stability of microbial food webs in the subalpine lake ecosystem, partial least squares path model (PLS-PM) structure equation models were constructed (the goodness of fit index value = 0.666, Fig. 5). Water temperature, pH, and bacterivorous protozoan components were the most important factors affecting the trophic interaction between bacterivorous protozoans and bacteria. Water nutrition and the composition of bacterivore protozoans were key factors significantly influencing bacterial richness. Also, the trophic interaction between bacterivorous protozoans and bacteria (path coefficient = 0.529, –0.812, *P* < 0.01) was the main factor significantly influencing microbial food web complexity and stability (Fig. 5). Additionally, positive simple linear regressions were found between Int and stability (Mod), whereas the complexity of L, LD, and C decreased with greater interaction (Fig. S8). These data suggest that predator‒prey interactions play important roles in determining the complexity and stability of microbial food webs.

**Fig 6:**
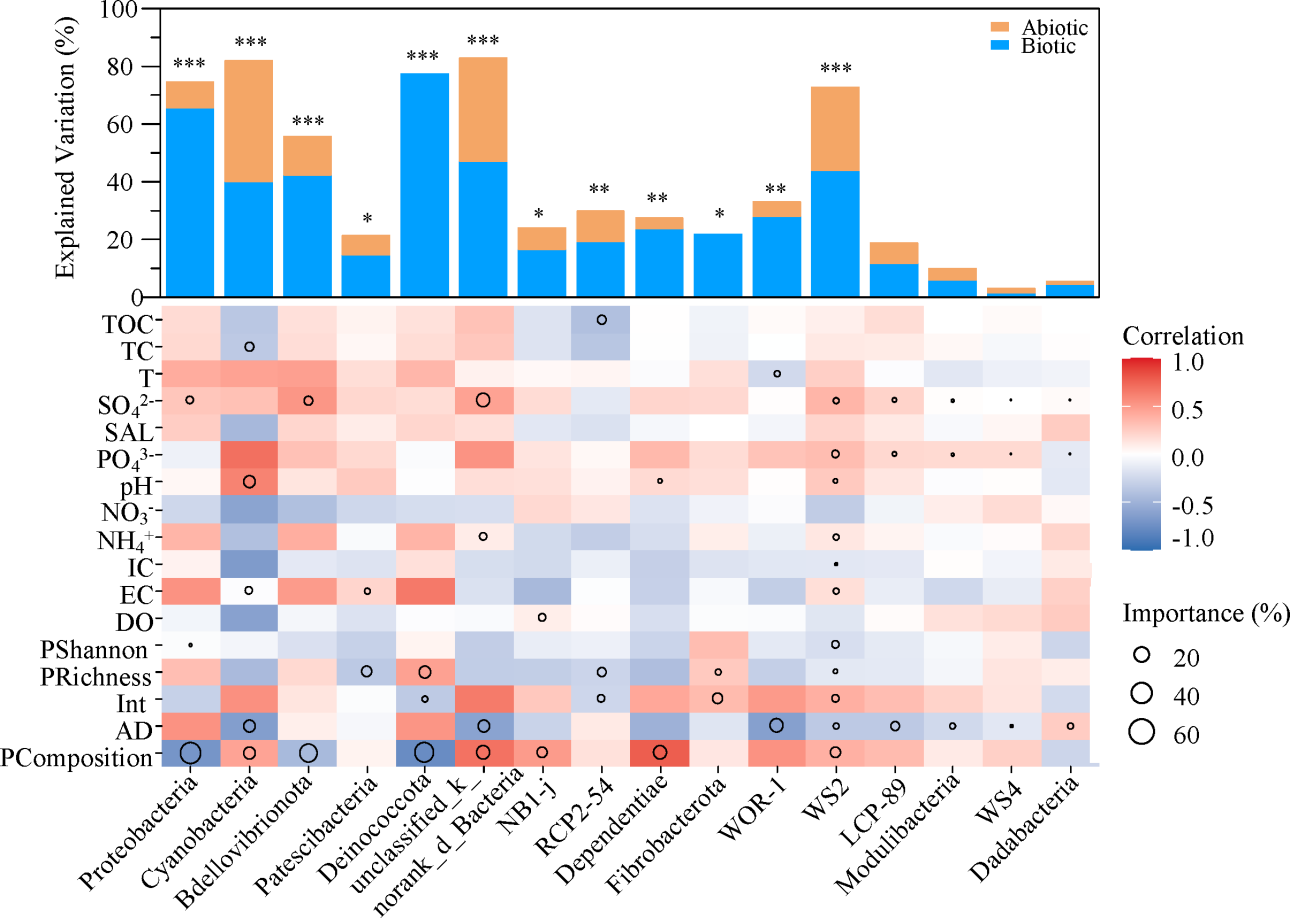
The relative contributions of biotic and abiotic factors to the abundance of the 17 bacterial phyla. Histograms show the *R* ^2^ values of the stepwise regression analysis for the major bacterial taxa (genera) (*P < 0.05 **P < 0.01, and ***P < 0.001). Circle size indicates the variable’s importance. A shade of color represents the strength of the Spearman correlation. PRichness, the OTU richness of bacterivorous protozoan communities; PShannon, the OTU Shannon index of bacterivorous protozoan communities; PComposition, PCoA1 of bacterivorous protozoan communities.

To further analyze which species members in the bacterial community interacted with bacterivorous protozoans, we quantified the contributions of the factors to each bacterial phylum by using multiple regression models and variance decomposition analysis. The results showed that the bacterivorous protozoan composition was the best predictor of the relative abundance of 17 bacterial phyla (Fig. 6). Overall, these results demonstrated that the trophic interaction between bacterivorous protozoans and 17 specific bacterial phyla played a vital role in the complexity and stability of microbial food webs in subalpine lakes.

## DISCUSSION

### Response of the diversity and structure of protist communities to seasonal changes

Our results suggested that the alpha diversity of the protist community was highest in autumn and lowest in spring (Fig. 1A). These results are not consistent with previous research suggesting that protist diversity was higher in spring and autumn and lower in summer and winter (21). This discrepancy may be explained by variations in environmental factors such as temperature, pH, DO, SAL, and nutrients (TOC, NH_4_^+^-N, and TC) (Fig. S2A) in the subalpine lake. Additionally, the trophic interactions between organisms (e.g., parasitism and symbiosis) are the main factors driving the diversity of planktonic protists in the ocean, which provides a greater understanding of the contribution of protists to the marine ecosystem (34). The distribution pattern of the protist community significantly changed (*P* < 0.05) (Fig. 1B). Moreover, its distribution was affected by many factors, especially SAL, NH_4_^+^-N, and temperature (Fig. S2B). In line with this result, environmental parameters directly or indirectly affect the structure of protist communities in aquatic ecosystems (35). For example, the distribution of protists in the coastal intertidal zone has a distinct biogeographical pattern, while the community structure is mainly affected by water temperature, SAL, and nitrogenous nutrients (36). Considerable evidence (37, 38) indicates that biological interactions (e.g., herder communities or parasites) might also influence the structure of protist communities, which could also be another driving force. Additionally, experimental studies that include more unmeasured environmental and biological factors are needed to obtain more accurate results for lake protection and resource planning. Collectively, these results revealed that the diversity, community structure, and community composition of protist communities showed significant seasonal differences and were mainly influenced by environmental factors such as water temperature and SAL in the studied subalpine lake.

### Response of the composition and structure of protistan functional communities to seasonal changes

Our data indicated that the bacterivores of protistan functional groups were dominant in different seasons (Fig. 2A). Our data support previous studies stating that predatory protists were the more dominant functional communities in a variety of habitats, including freshwater (39), marine (39), city water (26), agricultural soil (40), and rhizosphere soil (41). Our study also found that the Shannon index (Fig. 2B) and PCoA showed significant differences in protistan functional communities (algivores, bacterivores, parasites, nonselective omnivores, phototrophs, and raptors) in all seasons (Fig. 2C). The structures of the protistan functional communities were limited by environmental factors such as temperature, pH, and NH_4_^+^-N (Fig. S5). Moreover, the Mantel test showed that the correlation with temperature was the largest, which may be related to seasonal variations (Fig. S5). Previous studies have shown that protistan consumers (predatory), parasites, and phototrophs responded differently to environmental factors in the Swiss Alps (42).

Furthermore, most protistan predators (algivores, bacterivores, nonselective omnivores, and raptors) were significantly influenced by seasonal changes at the genus level; therefore, our results show that the dominant groups varied with season (Fig. S6). In the oligotrophic waters of the Fram Strait, ciliates were found to be mixotrophic (43). Furthermore, the presence of several potentially mixotrophic groups of protistan predators (e.g., *Cercomonas*, *Litostomatea_XXX*, and *Centroheliozoa_XXXX*) in this study revealed that mixotrophy was also important in different seasons and could contribute to the overall productivity of these waters (43). Cercozoans (Rhizaria) and ciliates (Alveolata) dominated among the predators (Fig. S4A, B, E, and F; Table S2), while bacterivorous protozoans accounted for 45%–78% (Fig. S4B) and were also documented in most eukaryotic groups (44). Protozoa feed selectively on bacteria at the genus level or even the species level (45). Therefore, protist predation plays a vital role in transferring carbon and energy to higher trophic levels as well as in releasing dissolved nutrients to the base of microbial food webs (46). In summary, protistan functional communities exhibited obvious seasonal dynamics in the subalpine lake, with temperature being the main driving force affecting the structure of the protistan functional communities.

### Response of the structure of microbial food webs to seasonal changes

Our data suggested that the microbial food webs in summer displayed higher L, LD, and C, which implied a more complex structure (Table S8). These results support previously reported data that the complexity of food webs increases with LD and C. More productivity in the summer is expected to promote microbial food web complexity. A high CC in summer and winter indicated that there were dense sub-groups of species interacting with one another (47). The most connected species in the genus *Cercomonas* had the widest ecological niches in summer and winter (Fig. 3) since they were generalists and omnivores (47). A greater number of species and longer food chains can be sustained, which correlates positively with omnivory, in which species have a higher chance of encountering prey in various habitats (13).

Notably, microbial food webs were significantly more stable in autumn (lower QSS) (Fig. 4D), indicating that they had a greater probability of recovery after perturbations (e.g., local loss of species) than in the other seasons (13). Compared with the other seasons, the lower stability of microbial food webs in summer might be due to their higher complexity. Ecological models indicate that complexity usually destabilizes food webs (48). Our result was supported by previous research, suggesting that stability decreases with increasing trophic levels and omnivory increasing (49). Food webs that include many generalist species are more vulnerable to perturbations since they frequently cause secondary extinctions (13). This is because generalists have many weak interactions that are important for stability (13). Omnivores can rapidly adapt to wider environmental conditions by changing their foraging habits to feed on the most abundant prey (50). A higher percentage of omnivory in summer and winter (31% and 31%, respectively) than in spring and autumn (26% and 28%, respectively) indicates that the network may be more robust to changes in prey abundances (Fig. 4C). The strength of species interactions influences the effects of omnivory on stability and local stability measures (e.g., QSS) (51). Thus, a comprehensive assessment of this impact should require a better understanding of the distribution of interactions’ strength. Taken together, these results indicate that the structure of microbial food webs in the subalpine lake showed clear seasonal patterns, with the most complex network in the summer exhibiting the lowest stability.

### Factors affecting the complexity and stability of microbial food webs

Our analysis indicated that the richness and composition of bacterivorous protozoans were important predictors influencing the complexity and stability metrics of microbial food webs (Fig. S7). This may be because the predation characteristics of bacterivorous protozoa depend on factors such as the size of the bacteria, its surface properties, and soluble secondary metabolites (e.g., type of terpene volatiles) (23). Protists contribute to the transfer of microbial biomass to higher trophic levels by predating on bacteria and fungi (18). Our study found that biotic factors represented by the trophic interaction of bacterivorous protozoans and bacteria significantly affected the complexity and stability of microbial food webs (Fig. 5). One possible reason for this is that species interactions of a community create a positive complexity–stability relationship within the community (52). Additionally, protist predation changes the bacterial community structure (53), stimulating bacterial activities in soil ecosystems (54). Previous studies have reported that the stability of meta-food webs is enhanced by far-ranging generalist top predators (14). Our results confirmed that predator and prey interactions were positively correlated with the stability metrics of microbial food webs (Mod) (Fig. S8).

The results showed that a biotic factor (the composition of protozoan communities) explained more variation in the abundance of 17 bacterial phyla (e.g., Proteobacteria and Cyanobacteria) than abiotic factors (Fig. 6). Confirming the results of a previous study, protist groups and the major bacterial groups (i.e., Cyanobacteria, Bacteroidetes, Proteobacteria, and Firmicutes) were involved in microbial interactions (predation) (44). Other studies have reported that bacteria are selectively preyed upon by protists at the phylum, genus, and species levels (55). Therefore, the bacterial species serving as protist prey significantly decrease (25), while other bacterial species might benefit from protist predation since the biomass of preyed-upon bacteria releases nutrients and decreases competition (45, 53), leading to changes in the bacterial community. Overall, predation has a great impact on the complexity and stability of microbial food webs in subalpine lakes.

### Conclusions

Our data suggest that the structure and diversity of protist communities and functional groups showed significant seasonal differences due to hydrologic (temperature and pH) and nutrient factors (NH_4_^+^-N) in a subalpine lake. The structure of microbial food webs was distinct among seasons, with the most complex network (in summer) exhibiting the lowest stability. The interaction of bacterivorous protozoans and the main bacterial phyla (i.e., Proteobacteria and Cyanobacteria) influenced the complexity and stability of microbial food webs. This study provides new insights into how the microbial food webs in lake ecosystems maintain their seasonal patterns, complexity, and stability.

## MATERIALS AND METHODS

### Study sites, sample collection, and environmental information

Gonghai Lake (38.91°N and 112.23°E) is in Ningwu County, Shanxi Province, China (Fig. S1). The lake is one of the field observation sites of the Shanxi Subalpine Grassland Ecosystem Field Observation and Research Station of the Ministry of Education. The lake is a hydrologically closed basin, and precipitation is the main source of water. Approximately 490 mm of precipitation falls each year in the research area, and an average temperature of 6.2°C is recorded every year (56).

Thirty-six water samples were collected at the center of the lake in May 2021 (spring), August 2021 (summer), October 2021 (autumn), and January 2022 (winter). Each water sample was prefiltered through a 200-µm mesh. Then, approximately 2 L was filtered through a 0.22-µm pore size filter (Millipore, Jinteng, Tianjin, China). The filters were then preserved at −80°C for DNA extraction.

Environmental variables (Table S1) such as electrical conductivity (EC), temperature (T), salinity (SAL), pH, dissolved oxygen (DO), ammonium (NH_4_^+^-N), and nitrate (NO_3_^−^-N) content were monitored at the sampling site using a portable water multiparameter quality monitor (Aquaread AP-2000, England, United Kingdom). Total organic carbon (TOC), total carbon (TC), and inorganic carbon (IC) were analyzed using a TOC analyzer (Shimadzu, TOC-VCPH, Shimane, Japan); sulfate (SO_4_^2−^-S) and phosphate (PO_4_^3−^ -P) were measured using an automated discrete analyzer (DeChem-Tech., CleverChem380, Hamburg, Germany).

### DNA extraction, Illumina sequencing, and bioinformatics analysis

Total DNA was extracted from the nitrocellulose filter membranes according to the steps of the FastDNA Spin Kit for Soil (MP Biomedicals, USA). DNA was quantified using a NanoDrop ND-2000 spectrophotometer (Thermo Fisher Scientific Inc., USA). Then, the extracted DNA was assessed by 1% agarose gel electrophoresis and stored in a freezer at −80℃ until amplification and sequencing. The V4 region of the 18S rRNA gene was amplified using the primers TAReuk454FWD1F (5′-CCAGCASCYGCGGTAATTCC-3′) and TAReukREV3R (5′-ACTTTCGTTCTTGATYRA-3′). The primers 338F (5′-ACTCCTACGGGAGGCAGCAG-3′) and 806R (5′-GGACTACHVGGGTWTCTAAT-3′) were used to amplify the V3-V4 region of the 16S rRNA bacterial gene. The PCR products were sequenced using the MiSeq platform (Illumina Inc., USA) at Shanghai Majorbio Biopharm Technology (Shanghai, China).

The raw sequencing data were generally filtered using QIIME2, and the chimeric sequences were identified and removed using USEARCH. Sequences with 97% sequence similarity were clustered into operational taxonomic units using UPARSE v7.0.1090 (57). Bacterial and protist OUT taxonomic information was classified by comparing the representative sequences of OUTs with the Silva 138 database and the PR^2^ (4.14 version) database (58). Sequencing reads of fungi, Rhodophyta, Streptophyta, and Metazoan were removed to focus on protist communities (59). The trophic functional groups of the protistan communities, including algivores (A), bacterivores (B), mycophagous (M), nonselective omnivores (N), heterotrophic parasites (H-P), phototrophs (P), raptors (R), saprotrophs (S), and unknown (U), were assigned based on existing literature (60) and the Protist Interaction Database (PIDA; https://doi.org/10.5281/zenodo.1195514) (see Table S2).

### Microbial food webs construction and topology

Network research provides a framework and tool for describing the structure of food webs (13). Food webs consist of trophic species, so we collected trophic interactions (prey‒predator) of genera in different seasons (Table S2). Trophic species are represented as nodes in the network. They can correspond to taxonomic groups at the species level (genus), organisms that share the same sets of predators and prey (e.g., algae, bacteria, and fungi), and non-living compartments of matter and energy (e.g., detritus and dissolved organic carbon) (10, 13). The trophic network in four seasons was defined by an adjacency matrix of pairwise interactions, in which each element *a*_ij_ = 1 when the j-genus preyed on the i-genus and *a*_ij_ = 0 otherwise (Tables S3 to S6). Microbial food webs are directed graphs of those matrices. We used a network approach to explore and compare the general structural properties of seasonal microbial food webs. The “multiweb” R package was applied to calculate all network metrics and food web simulations (10, 13). Microbial food web visualization was carried out using the Gephi interactive platform (https://gephi.org).

To evaluate the structure of seasonal microbial food webs, we calculated the following food web metrics: species, links, linkage density, connectance, mean trophic level, and omnivory (Table S7). L, LD, and C were used to assess the complexity of microbial food webs. The topologies of the microbial food webs were measured based on three properties: clustering coefficient (CC), modularity, and characteristic path length (Table S7). Additionally, the topological features of microbial food webs in each sample, including the average degree and the proportion of interaction associations between bacterivorous protozoans and bacterial taxa (Int), were estimated as potential biotic interactions (61).

### Structure and stability comparison of microbial food webs using a randomization algorithm

The curve-ball algorithm randomizes the network structure preserving the number of predators and prey for each species and their trophic interactions. A total of 1,000 network randomizations were performed for each microbial food webs, and we calculated the following structure and stability metrics in four seasons: mean trophic level, omnivory, modularity, and quasi sign-stability. If the empirical values of metrics were tested within the distribution of the simulated microbial food webs, we believed that simulations fitted the empirical values and could be used for comparisons. Then, the 95% confidence intervals were calculated, and the distributions obtained for stability metrics were compared by the two-sided Kolmogorov‒Smirnov test (10).

### Statistical analyses

The alpha diversity was estimated using the “vegan package” in R 4.1.3. A one-way analysis of variance (ANOVA) was conducted to examine statistically significant differences among the four seasons in the Shannon index and water physicochemical variables.

Principal coordinates analysis (PCoA) was performed using the “vegan package” (Hellinger transformed) to explore differences in protistan taxonomic and functional communities. In the subsequent analyses, the composition (PCoA1 and PCoA2) was used to represent the variation in bacterivore protozoan community compositions. Random forest was used to calculate the significance of the effect of factors (evaluated by diversity and the beta-PCoA axes of bacterivore protozoans) on the complexity and stability of microbial food webs using the “rfPermute” package (62).

Spearman correlation analysis was applied to analyze correlations between environmental parameters and the alpha diversity of protistan communities. To examine the effect of environmental factors on the protist community structure, distance-based redundancy analysis (db-RDA) was performed using the vegan package, and forward selection was determined using Monte Carlo permutation tests (permutations = 9999).

A partial least squares path model was constructed using the R package “plspm” to analyze the effects of abiotic (water property and nutrition) and biotic (composition and diversity) factors on the complexity and stability of microbial food webs (8). After removing the variables with loadings <0.7, we performed the final PLS-PM structure equation with the remaining variables (63). The path coefficients were used to quantify the relationships between these block variables. The prediction performance of the model was evaluated using the goodness of fit index (GoF) and *R* ^2^.

The correlations among the metrics (complexity and stability of microbial food webs) and Int (bacterial and bacterivorous protozoan taxa) were fitted via linear regression. The multiple regression model with variance decomposition analysis was used to estimate the importance of influencing factors for differences in the relative abundance of specific phyla by the lm and cacl.relimp functions.

## Data Availability

All the raw sequence data of the eukaryotic 18S rRNA gene and bacterial 16S rRNA were submitted to the NCBI GenBank (accession numbers PRJNA905214 and PRJNA905182).

## SUPPLEMENTAL MATERIAL

Supplemental figures spectrum.02411-23-s0001.docx Fig. S1 to S8.

Supplemental tables spectrum.02411-23-s0002.xlsx Tables S1 to S10.

ASM does not own the copyrights to Supplemental Material that may be linked to, or accessed through, an article. The authors have granted ASM a non-exclusive, world-wide license to publish the Supplemental Material files. Please contact the corresponding author directly for reuse.
