# Supplementary material for: Predation has a significant impact on the complexity and stability of microbial food webs in subalpine lakes
Source: Microbiol Spectr. 2023 Oct 3;11(6):e02411-23. doi: 10.1128/spectrum.02411-23 (PMC10714739; doi:10.1128/spectrum.02411-23)
Supplement: Supplemental figures — Fig. S1 to S8. [file spectrum.02411-23-s0001.docx]

**Supplementary materials**


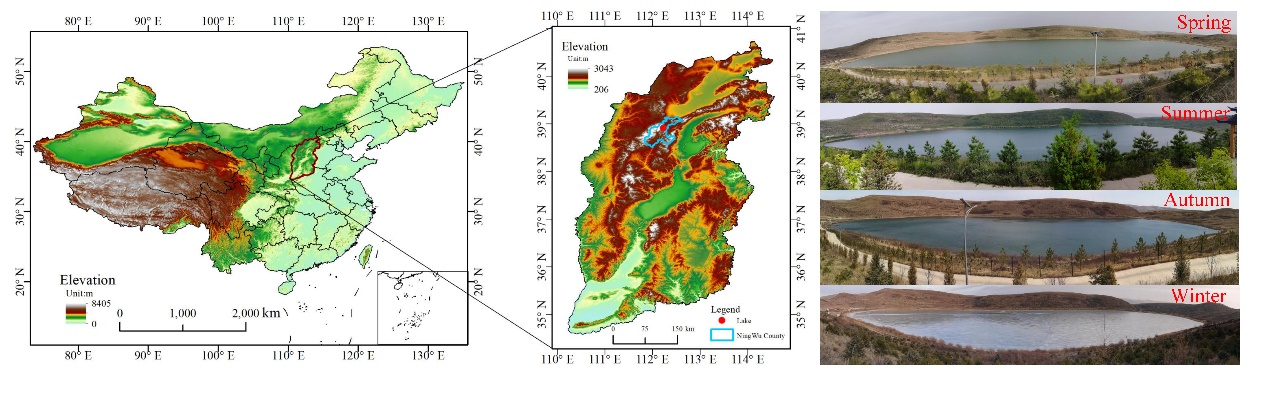


FIG S1 Map showing the location of sampling sites in the Ningwu County, Shanxi, China.


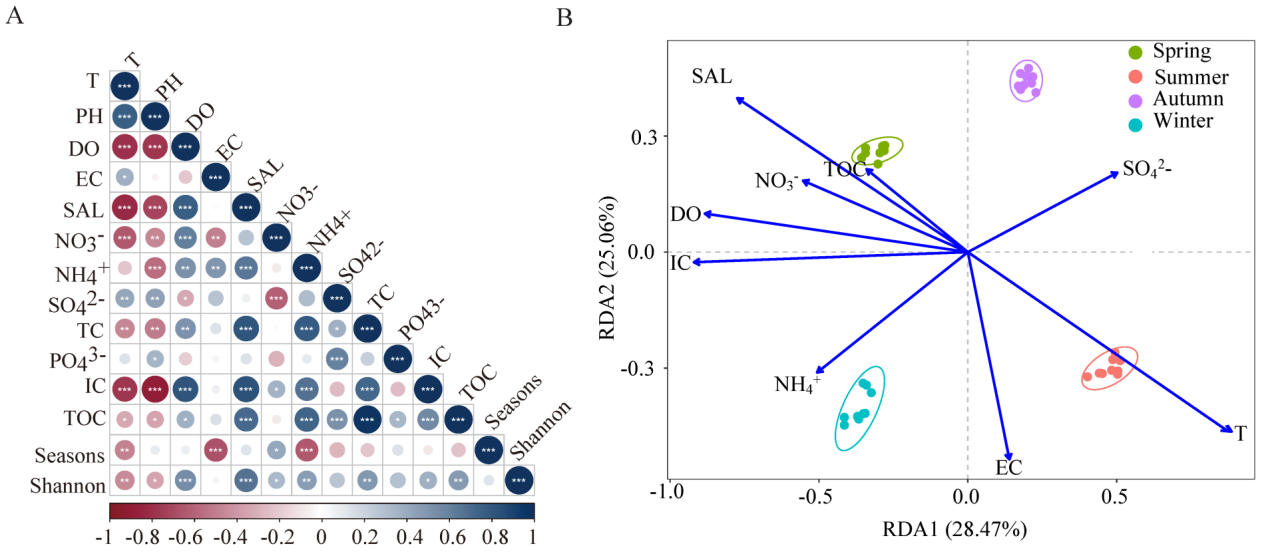


FIG S2 Driving factors of protists communities’ alpha diversity and compositions. (A) The Spearman correlations between environmental factors and the alpha diversity of protists communities. (B) Distance-based redundancy analysis (db-RDA) of protists communities with environmental variables.


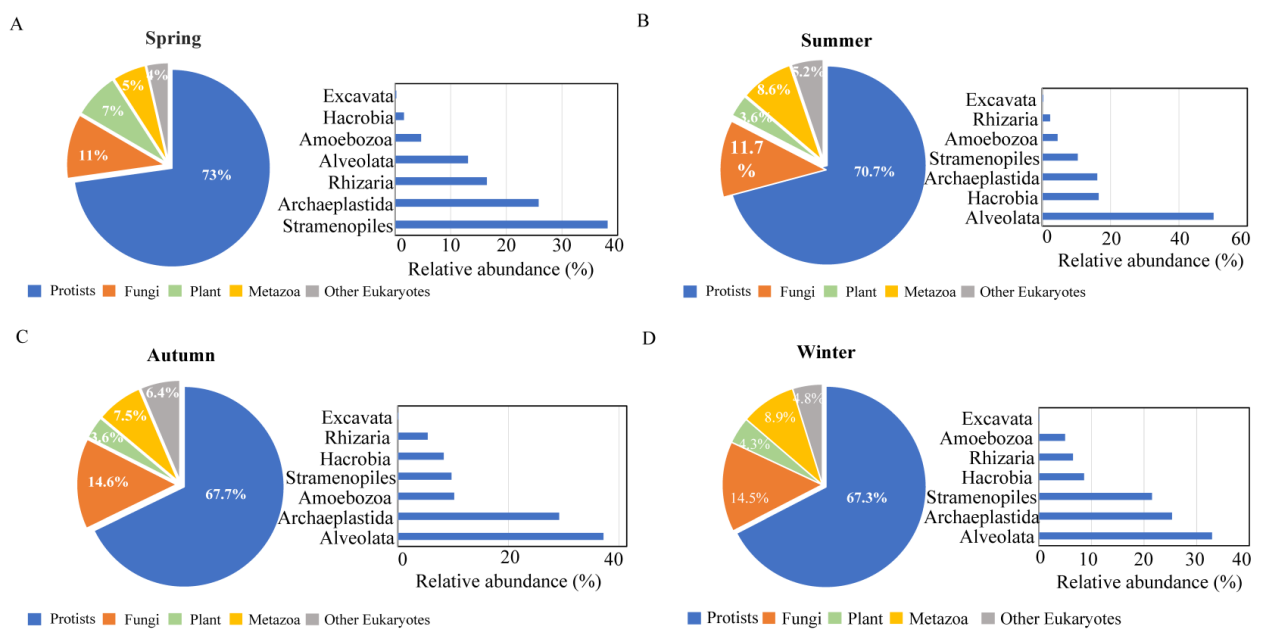


FIG S3 Compositions of eukaryotic communities across different seasons. The right panels present the relative abundances of protist supergroups. (A) spring, (B) summer, (C) autumn, and (D) winter.


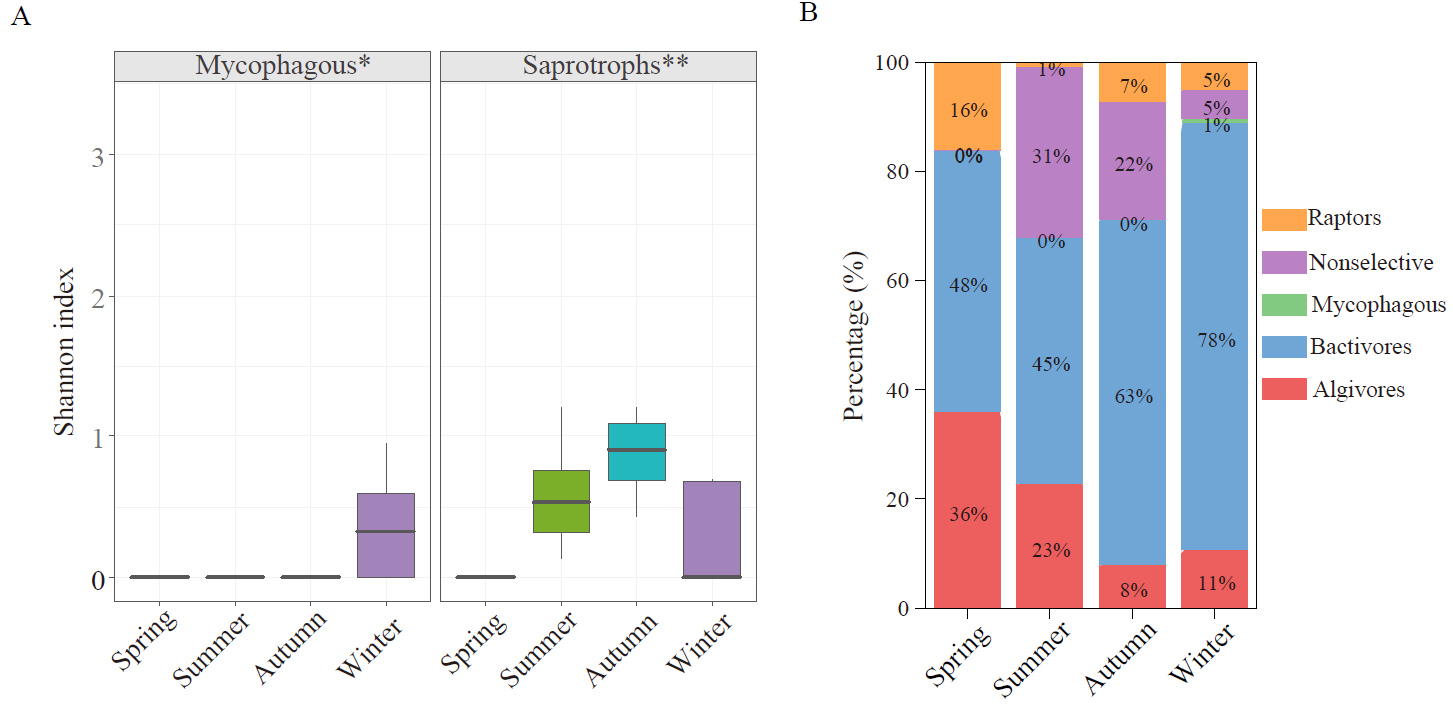


FIG S4 (A)Alpha diversity of mycophagous and saprotrophs protistan functional communities and (B) The percentage of predatory protists.


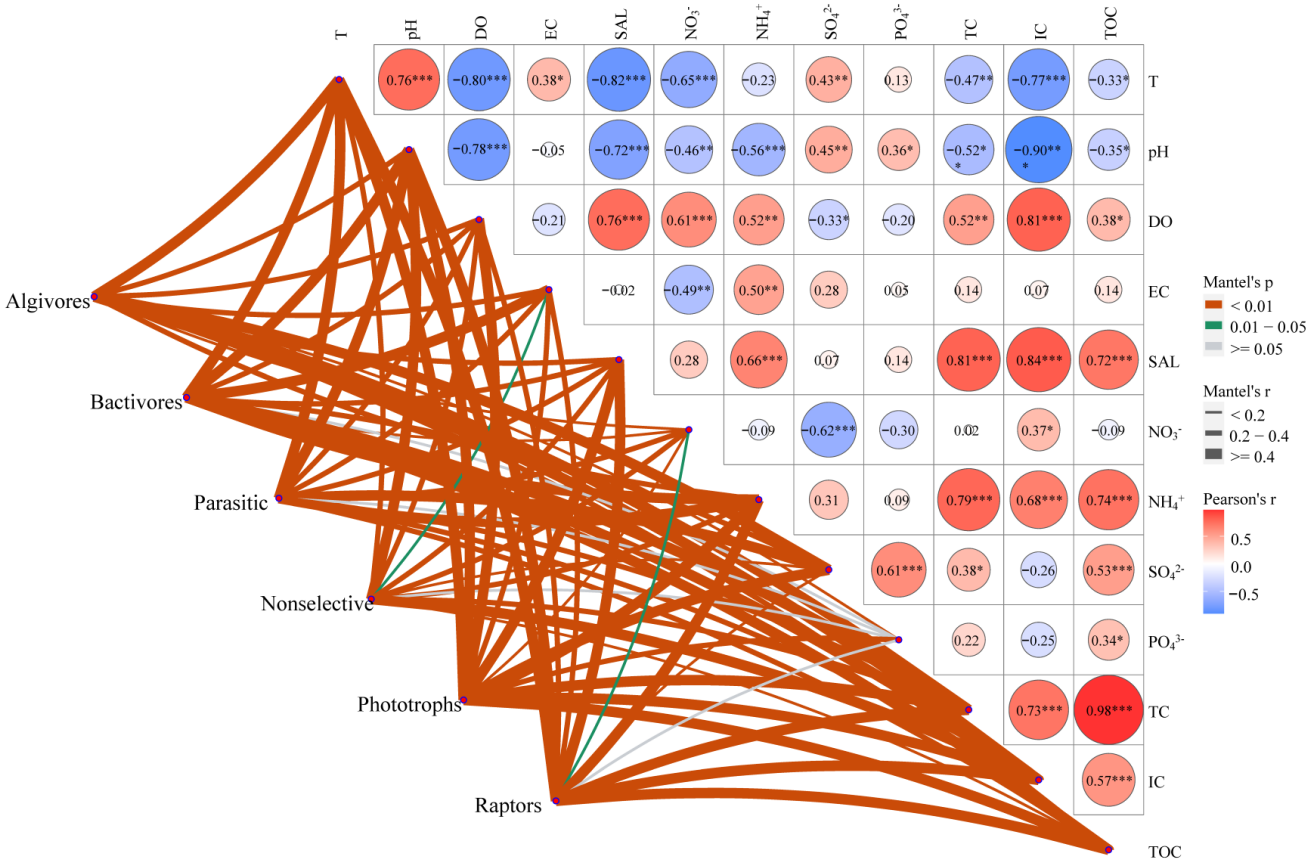


FIG S5 Driving factors of protistan functional communities’ composition. Line width represents the partial Mantel’s r statistic for the corresponding correlation, while line color incates that significances were tested based on 999 permutations. T, temperature; pH；Sal，salinity；DO, dissolved oxygen; EC, electrical conductivity; TC, total carbon; TOC, total organic carbon; IC, inorganic carbon; NH_4_^+^-N, ammonium nitrogen; NO_3_^-^-N, nitrate nitrogen; PO_4_^3-^-P, phosphate phosphorus; SO_4_^2^-S, sulfate sulfur.


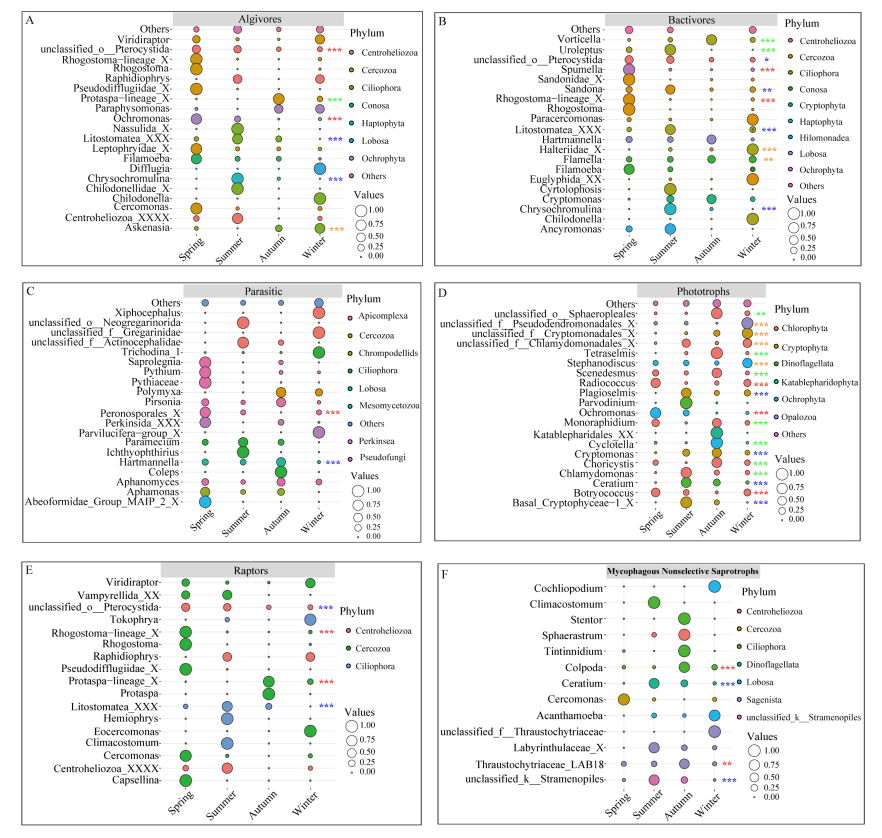


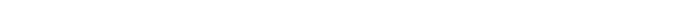
FIG S6 Genus level relative abundances of protistan functional communities, including (A) algivores, (B) bacterivores, (C) parasites, (D) phototrophs, (E) raptors and (F) mycophagous, nonselective omnivores, saprotrophs. *, **, or *** indicate significant differences based on a least significant difference test at *p* < 0.05, *p* < 0.01, and *p* < 0.001, respectively. Red, blue, green and orange asterisks (*, **or***) indicate that the level of the gene is enriched in the spring, summer, autumn, and winter, respectively.


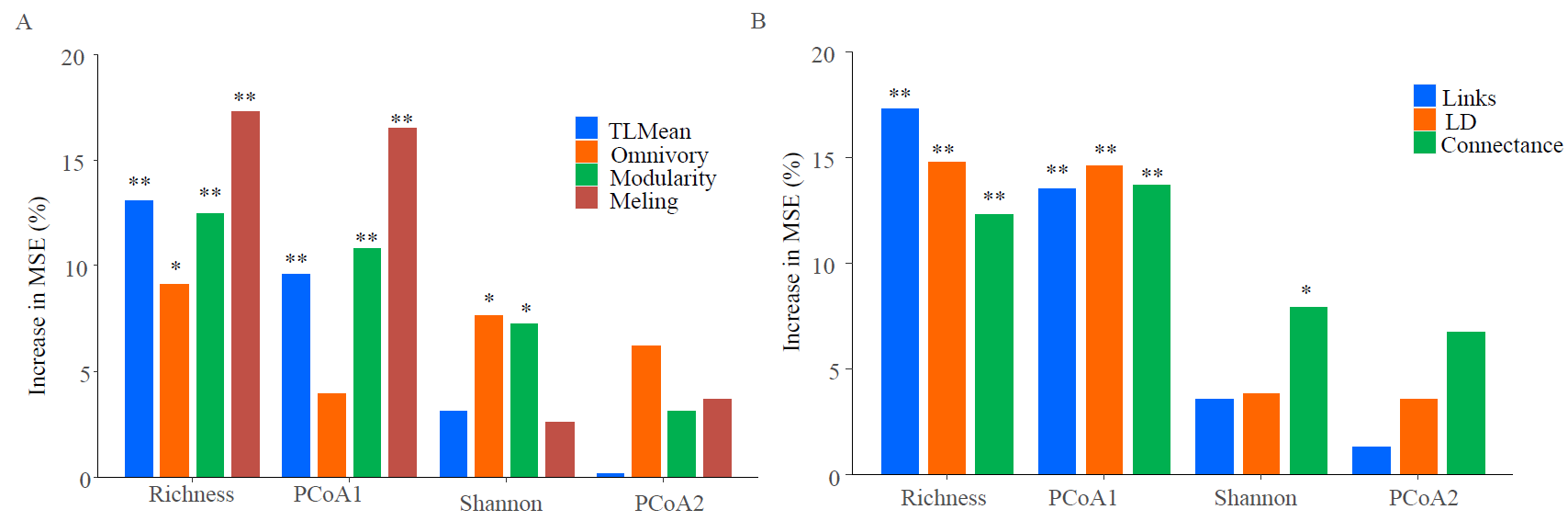


FIG S7 Random Forest mean predictor importance of the diversity (Richness and Shannon index) and composition (Beta-PCoA1 and PCoA2) of bacterivores protozoan communities for the (A) complexity and (B) stability metrics of microbial food webs.


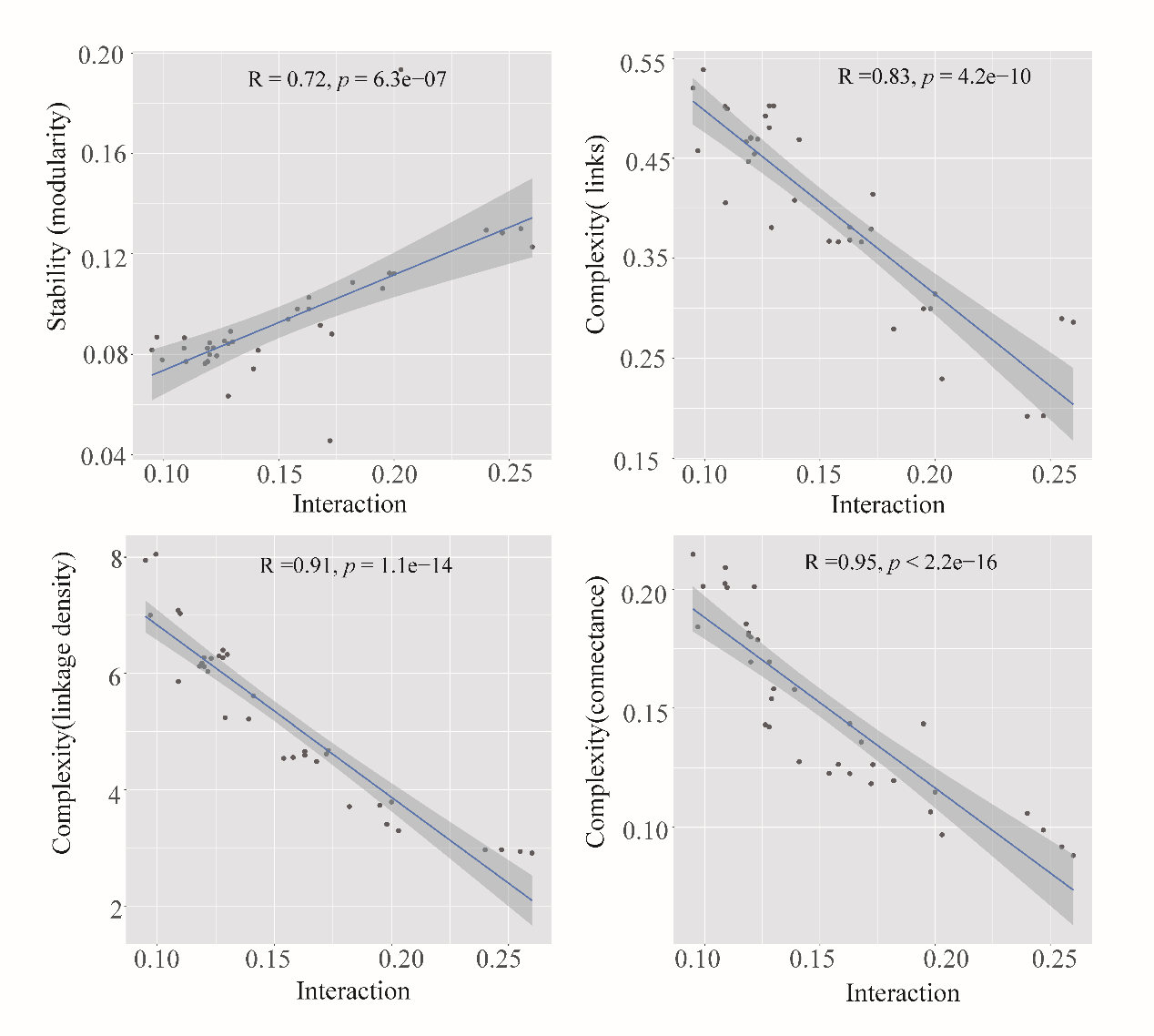


FIG S8 Correlation analysis of the relationship between the interaction and (A) modularity, (B) links, (C) linkage density, and (D) connectance of microbial food webs.
